# Modelling the free energy profile of the mitochondrial ADP/ATP carrier

**DOI:** 10.1016/j.bbabio.2017.05.006

**Published:** 2017-11

**Authors:** Roger Springett, Martin S. King, Paul G. Crichton, Edmund R.S. Kunji

**Affiliations:** Medical Research Council Mitochondrial Biology Unit, University of Cambridge, Cambridge Biomedical Campus, Wellcome Trust/MRC Building, Hills Road, Cambridge CB2 0XY, UK

**Keywords:** Adenine nucleotide translocase, Adenine nucleotide translocator, Substrate exchange, Mathematical model, Boltzmann distribution, Transport protein

## Abstract

The mitochondrial ADP/ATP carrier catalyses the equimolar exchange of adenosine di- and tri-phosphates. It operates by an alternating access mechanism in which a single substrate-binding site is made available either to the mitochondrial matrix or the intermembrane space through conformational changes. These changes are prevented in the absence of substrate by a large energy barrier due to the need for sequential disruption and formation of a matrix and cytoplasmic salt bridge network that are located on either side of the central cavity. In analogy to enzyme catalysis, substrate lowers the energy barrier by binding tighter in the intermediate state. Here we provide an *in-silico* kinetic model that captures the free energy profile of these conformational changes and treats the carrier as a nanomachine moving stochastically from the matrix to cytoplasmic conformation under the influence of thermal energy. The model reproduces the dependency of experimentally determined *k*_*cat*_ and *K*_*M*_ values on the cytoplasmic network *strength* with good quantitative accuracy, implying that it captures the transport mechanism and can provide a framework to understand the structure-function relationships of this class of transporter. The results show that maximum transport occurs when the interaction energies of the cytoplasmic network, matrix network and substrate binding are approximately equal such that the energy barrier is minimized. Consequently, the model predicts that there will be other interactions in addition to those of the cytoplasmic network that stabilise the matrix conformation of the ADP/ATP carrier.

## Highlights

• A Markov state model of molecular kinetics is developed for the ADP/ATP carrier.
• Captures the free energy profile of the conformational changes of transport
• Validated against kinetic measurements from a set of mutant carriers
• Establishes how structure shapes the free energy profile, which defines function

## 1 Introduction

In the functioning mitochondrion the mitochondrial ADP/ATP carrier forms part of the mitochondrial ATP circuit, importing cytosolic ADP into the matrix and exporting ATP synthesized by the F_o_F_1_ ATP synthase into the intermembrane space, which is confluent with the cytosol [1], see [2] for a recent review. The protein cycles between matrix and cytoplasmic conformations in which the substrates can bind reversibly from the mitochondrial matrix and the intermembrane space, respectively. The specific inhibitors atractyloside and carboxyatractyloside lock the transporter in the cytoplasmic conformation, whereas bongkrekic acid locks the carrier in the matrix conformation [3], [4], [5]. As with all mitochondrial carrier proteins, the ADP/ATP carrier consists of three homologous ~ 100 amino acid repeat domains [6], [7], each composed of two transmembrane α-helices separated by a matrix loop and small α-helix [8]. The atomic structures of the bovine and yeast ADP/ATP carriers inhibited by carboxyatractyloside show that the three repeat domains form a six α-helical barrel around a central cavity that is open to the intermembrane space [8], [9]. By considering conservation of amino acids as well as distance and chemical constraints, and by analysis of the pseudo-symmetry of mitochondrial carriers, a single substrate-binding site was identified in the centre of the cavity [10], [11], [12]. ADP also binds to this site in labelling studies [13] and molecular dynamics simulations [14], [15], [16].

Strict exchange of nucleotides is essential because the presence of the membrane potential (positive outside) would lead to their depletion from the matrix due to their negative charge if uniport activity were to occur. Furthermore, exchange obligates the carrier to act as an energy-transducing protein due to the charge difference between ATP^4 −^ and ADP^3 −^ and hence maintains the cytosolic ATP/ADP ratio higher than the mitochondrial ATP/ADP ratio [17]. Structural evidence supports an alternating access mechanism of substrate exchange by the carrier [9], [12], equivalent to a ping-pong mechanism, where one substrate is transported but leaves the substrate binding site before a counter-substrate binds for transport in the opposite direction. Importantly, for strict exchange in this manner, the change between matrix and cytoplasmic conformations should not occur in the absence of bound substrate. Klingenberg [18] suggested that there is a large energy barrier for conversion between the matrix and cytoplasmic conformations and that, in analogy to enzyme catalysis, the presence of bound substrate lowers this barrier by tighter binding in the intermediate conformation, but the molecular nature of this barrier has not been addressed.

The odd-numbered transmembrane α-helices (H1, H3 and H5) of the carrier each contain the signature motif of the carrier family, Px[*DE*]xx[RK] [6], [19], which forms an inter-domain salt bridge network on the matrix side of the protein, closing access of the substrate binding site to the matrix when the carrier is in the cytoplasmic conformation [8]. By using sequence analysis [12] a second motif was discovered on the even-numbered transmembrane α-helices with the consensus [FY][*DE*]xx[RK] and it has been shown that the charged residues of this motif form an inter-domain salt bridge network on the cytoplasmic side, closing access to the substrate binding site to the intermembrane space when the carrier is in the matrix-facing conformation [9], [20]. The conformational change allowing the sequential breaking and formation of these networks might involve movement around the conserved prolines and glycines [21] or domain motions [9].

**Fig. 1:**
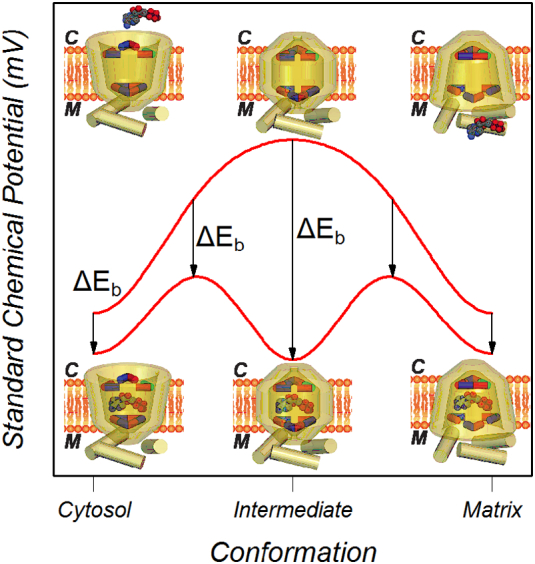
Proposed energy profile for the mitochondrial ADP/ATP carrier. From the unbound intermediate conformation, the free energy of the carrier is lowered by formation of the bonds of the matrix and cytoplasmic network on movement to the cytoplasmic or matrix conformation. Substrate can only bind from the matrix or cytoplasmic conformation but the binding energy is greater in the intermediate conformation lowering the energy barrier for transport.

The sequential breaking and formation of the bonds of the matrix and cytoplasmic networks provides an energy barrier for the change between cytoplasmic and matrix conformations. Fig. 1 shows a plausible energy profile for the carrier based on this mechanism. In the absence of substrate, the changes between cytoplasmic and intermediate conformations would break the bonds of the matrix salt-bridge network, raising the standard chemical potential of the carrier to a maximum in the intermediate conformation. Further changes towards the matrix conformation would form the bonds of the cytoplasmic salt-bridge network, lowering the standard chemical potential, and making the substrate-binding site accessible from the matrix side. Binding of substrate in the cytoplasmic conformation lowers the standard chemical potential of the carrier due to the energy of the bonds (*ΔE*_*b*_) formed between the carrier and the substrate. The conformational changes would then allow the substrate to bind tighter, increasing the binding energy until it was at a maximum in the intermediate-state. Further conformational changes towards the matrix-state would weaken the substrate-carrier bonds and lead to a decrease in the binding energy. Thus tighter binding of the substrate in the intermediate conformation would lower the energy barrier between cytoplasmic and matrix conformations.

The magnitude of the energy barrier between cytoplasmic and intermediate conformations, and between matrix and intermediate conformations, will depend on the total number and individual strengths of the salt bridges in the matrix and cytoplasmic networks, respectively. The matrix network of the wild type carrier contains three salt bridges, but is further strengthened by a glutamine residue that forms a brace between residues of the salt bridge linking domains one and three [9]. This gives the matrix network a strength equivalent to 3.5 salt bridges, assuming that a hydrogen bond has about half the strength of a salt bridge [12]. There is no structure of the matrix state, but on the basis of sequence information, the cytoplasmic network of fungal carriers contains two salt bridges and a hydrogen bond, giving it a relative strength of 2.5, whereas that of the mammalian carriers contains three salt bridges, providing an overall strength of 3.0. Recently, we have generated a set of mutants in the fungal ADP/ATP carrier in which the number of salt bridges, and hence interaction energy, of the cytoplasmic network was increased and decreased. We have shown that the stability of the carrier in detergent is independent of the cytosolic network strength when the carrier is locked in the cytoplasmic conformation with carboxyatractyloside, but is proportional to the network strength when the carrier is locked in the matrix conformation with bongkrekic acid, demonstrating that the cytoplasmic network is only interacting in the matrix conformation [20].

Previous mathematical models have treated the intermediate conformation as a transition state between matrix and cytoplasmic conformations [22], [23], [24] and did not treat the carrier as a nanomachine that moves stochastically and continuously from the matrix to cytoplasmic conformation under the influence of thermal energy [25]. In these models the rate constants for this conformational change, which determines the transport rate, must be fitted from experimental data. Here we have developed a Markov model of molecular kinetics [26] that captures the stochastic motion of the carrier and, with the addition of a substrate binding site, can predict transport rates based on the free energy profile of the conformational changes that occur, which in turn is determined by the strength of substrate binding and the two salt bridge networks. The model is validated against kinetic measurements made with a set of mutant carriers with altered cytoplasmic network strengths and is then used to establish how the free energy profile, which is determined by the structure, defines the function of the carrier.

## 2 Methods

**Fig. 2:**
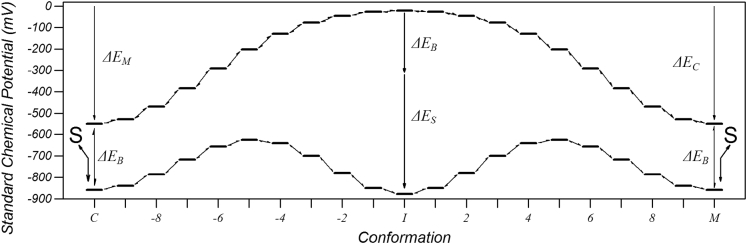
A summary of the kinetic model. Horizontal lines represent states and bidirectional arrows represent reactions. The zero on the energy scale is set for the carrier with no substrate bound and the networks disengaged. The chemical potential of the unbound cytoplasmic conformation (C) is lowered by formation of the matrix network with free energy *ΔE*_*M*_. Likewise, the matrix conformation (M) is lowered by formation of the cytoplasmic network with free energy *ΔE*_*C*_. The bound states are further lowered by the free energy of binding *ΔE*_*B*_ in the cytoplasmic and matrix conformation in the absence of the induced fit and the conformation-dependent induced-fit binding energy *ΔE*_*S*_ such that the total binding energy is in a given conformation is *ΔE*_*B*_ + *ΔE*_*S*_.

The continuous change between matrix and cytoplasmic conformations can be considered as a diffusion along a one-dimensional track by random walk governed by thermal energy [25] and was modelled as 21 discrete steps labelled *c* = − 10 to + 10 where *c* = − 10 is the cytoplasmic conformation, *c* = 0 is the intermediate conformation and *c* = + 10 is the matrix conformation. The model includes a single substrate binding site that could be either empty or have a nucleotide bound, thus the state < c,s > of the system is fully described by the conformation, c, and the occupancy, s, of the substrate binding site. The kinetic model is summarized in Fig. 2 where the horizontal lines represent the states and bi-arrows represent transitions, analogous to chemical reactions, which connect pairs of states with forward and reverse rate constants. Binding of a substrate from the respective compartment was only allowed from the cytoplasmic and matrix conformation with rate constants *k*^*c* , *e* →* c* , *s*^ and *k*^*c* , *s* →* c* , *e*^ where *c* = − 10 or + 10, *e* represents the unbound carrier and *s* represents the bound nucleotide. The conformational changes were modelled by connecting neighbouring conformations *c* and *c* + *1* with forward and reverse rate constants, *k*^*c* , *s* →* c* + 1 , *s*^ and *k*^*c* + 1 , *s* →* c* , *s*^ which depend on the substrate, s, bound (s can be either none or a nucleotide). The total number of states in the system is 21(N + 1) and the number of reactions is 20(N + 1) + 2 N were N is the number of substrates that can bind to the carrier in a mutually exclusive way. Only a single chemical species, ADP, was considered as the model is compared to published experimental data in which radio-labelled cytosolic ADP was exchanged for unlabelled matrix ADP [20]. This data was modelled using two substrates (*N* = 2), ‘labelled’ and ‘unlabelled’ ADP, so that substrate that bound from one side and then was released to the same side could be distinguished from substrate that was transported, i.e. bound from one side and released to the other side. The same rate constants were used throughout for both labelled and unlabelled substrate, as they are considered identical chemical species.

The standard free energy change for the enzyme with substrate *s* bound between neighbouring conformations *c* and *c* + *1*, *ΔG*_*c* , *s* →* c* + 1 , *s*_^0^, is related to the rate constants and standard chemical potentials of states <* c,s >* and <* c* + *1,s >*, *μ*_*c* , *s*_^0^ and *μ*_*c* + 1 , *s*_^0^, respectively, by

(1) $$\Delta G_{c,s\to c+1,s}^{0}=-k_{B}T\mathrm{Ln}\left(\frac{k^{c,s\to c+1,s}}{k^{c+1,s\to c,s}}\right)=\mu_{c+1,s}^{0}-\mu_{c,s}^{0}$$

Where *k*_*B*_ is the Boltzmann's constant and *T* is the temperature in Kelvin. Free energies are expressed in mV (10.36 mV = 1 kJ/mol) so that the thermodynamic efficiency of the carrier can ultimately be compared with that of the electron transport chain [27]. The modelling assumed a temperature of 37 °C at which *k*_*B*_*T* has a value of 26.7 mV.

The standard chemical potential of the carrier in the *c*^*th*^ conformation with substrate, *s*, bound was assumed to be the sum of the free energy of formation of the matrix and cytoplasmic networks and substrate binding energy,

(2) $$\mu_{c,s}^{0}=\Delta E_{M}^{c}+\Delta E_{C}^{c}+\Delta E_{B}^{c,s}$$

where *ΔE*_*M*_^*c*^ and *ΔE*_*C*_^*c*^ are the free energy of the formation of the bonds of the matrix and cytoplasmic networks in the *c*^*th*^ conformation and are independent of substrate bound, respectively, and *ΔE*_*B*_^*c* , *s*^ is the free energy of substrate, s, binding in the *c*^*th*^ conformation, which is zero when no substrate is bound. To ensure the thermodynamic fidelity of the model, the rates were calculated from the standard free energy change of the reaction step by splitting it equally between the forward and reverse rates,

(3) $$\begin{array}{l} k^{c,s\to c+1,s}=k_{c}e^{-½\Delta G_{c,s\to c+1,s}^{0}/K_{b}T}\\ k^{c+1,s\to c,s}=k_{c}e^{½\Delta G_{c,s\to c+1,s}^{0}/K_{b}T} \end{array}$$

where *k*_*c*_ is the rate of the conformational change at *ΔG*^*0*^ = 0. The large number of discrete conformations ensured that the *ΔG*^*0*^ between any two neighbouring conformations was small so that the forward and reverse rates were similar to *k*_*c*_. The dependence of the interaction energy on conformational state was chosen to be Gaussian such that:

(4) $$\begin{array}{l} \Delta E_{M}^{c}=\Delta E_{M}e^{-{\left(\left(c+10\right)/5\right)}^{2}}\\ \Delta E_{C}^{c}=\Delta E_{C}e^{-{\left(\left(c-10\right)/5\right)}^{2}}\\ \Delta E_{B}^{c,s}=\Delta E_{S}e^{-{\left(c/4\right)}^{2}}+\Delta E_{B} \end{array}$$

where *ΔE*_*B*_ is the substrate binding energy in the cytoplasmic and matrix conformations in the absence of the induced-fit, Δ*E*_*C*_, Δ*E*_*M*_ and Δ*E*_*S*_ are scaling factors describing the strength of the cytoplasmic network, the matrix network and the induced-fit substrate binding energy, respectively. Note that the matrix network is engaged in the cytoplasmic conformation and so affects the chemical potential of the cytosolic conformation, and vice versa. This form of parameterization allows the conformation changes to be modelled with only four parameters: Δ*E*_*M*_, Δ*E*_*C*_ and Δ*E*_*B*_ and *k*_*c*_, and the complete model with only a further two parameters, *k*_*f*_^*s*^ and *k*_*r*_^*s*^, which are the forward and reverse rate constants for substrate binding.

The forward rate for substrate binding was assumed to be first order in substrate concentration such that the substrate binding energy is given by:

(5) $$\Delta E_{B}=-k_{B}T\mathrm{Ln}\left(\frac{k_{f}^{s}\left[S\right]}{k_{r}^{s}}\right)=-k_{B}T\mathrm{Ln}\left(\frac{\left[S\right]}{K_{d}^{s}}\right)=\Delta G_{B}^{0}-k_{B}T\mathrm{Ln}\left(\left[S\right]\right)$$

where *K*_*d*_^*s*^ is the disassociation constants for substrate, *s*, in the absence of induced-fit binding and *ΔG*_*B*_^0^ is the standard change in free energy of binding.

To obtain parameters for the model, experimental data for the mitochondrial ADP/ATP carrier of the thermophilic fungus *Myceliophthora thermophile* were used [20]. In brief, the carrier was expressed in *Lactococcus lactis* without the his-tag, membranes were fused with liposomes and transport parameters determined by uptake of ^14^C-labelled ADP [20]. The measured *k*_*cat*_ of the wild type carrier was approximately 40 ADP/s (table S1). The substrate binding rates are expected to be much faster than the transport rates [23] and molecular dynamics simulations report that ADP binds in ≈ 40 ns [15] hence the forward rate was set to 25 ns^− 1^ M^− 1^ giving a mean binding time of 40 ns at a concentration of 1 mM. A *K*_d_ of 40 μM was chosen so that the modelled *K*_*M*_ of transport matched the experimentally determined *K*_*M*_ of transport for weak cytoplasmic networks (Fig. 6) and this fixed the reverse rate constant to 1 × 10^6^ s^− 1^ (Eq. (5)). It was found that the transport was almost linear with the conformational change rate constant *k*_*c*_, which was set to 5.66 × 10^6^ s^− 1^ so that the model matched the measured transport rates when *ΔE*_*C*_ and *ΔE*_*B*_ were − 550 mV (Fig. 6). Simulations were carried out either by assembling the chemical master equation and solving it in the steady state by matrix inversion, or stochastically using Gillespie's algorithm [28] using software compiled with Delphi 2010 (Embarcadero Technologies).

## 3 Results

The breaking and formation of the bonds of the cytoplasmic and matrix networks produce an energy barrier to prevent the carrier switching between matrix and cytoplasmic conformations in the absence of substrate binding. The ability of this energy barrier to limit the rate of conformational change was explored by running simulations in the absence of an imposed substrate binding energy when the cytoplasmic and matrix network strengths were varied, but set equal to one another.

In order to distinguish between ADP that (1) bound from the cytoplasmic side and was released on the matrix side, (2) bound from the cytoplasmic side and was released to the cytoplasmic side, (3) bound from the matrix side and was released to the matrix side and (4) bound from the matrix side and released on the cytoplasmic side, simulations were carried out with “labelled” and “unlabelled” ADP, with both having identical rate constants.

**Fig. 3:**
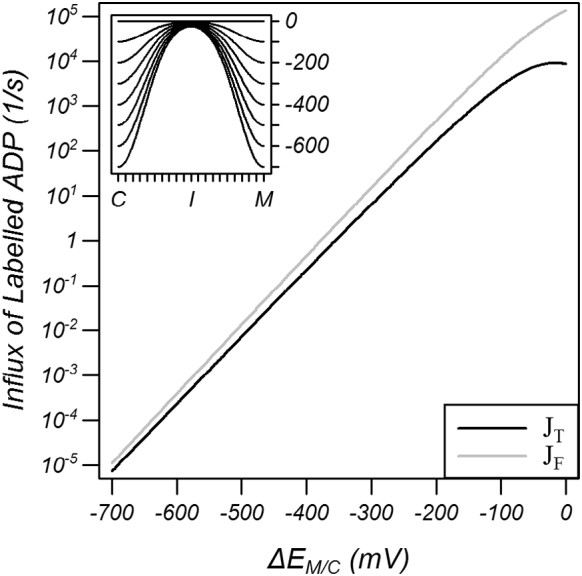
Transport (*J*_*T*_) as a function of the network strength in the absence of a substrate binding energy. Simulations were carried out with 5 mM of labelled ADP on the cytoplasmic side and 5 mM of unlabelled ADP on the matrix side with the efflux of unlabelled ADP plotted as the cytoplasmic and matrix network strengths were varied equally (*ΔE*_*C*_ = *ΔE*_*M*_ = *ΔE*_*M/C*_). The inset shows representative energy profiles. J_F_ is an estimation of the limiting forward rate calculated with a simple model (see Discussion).

In line with thermodynamics principles, there is no net transport of ADP in the steady state when substrate concentrations were set to 5 mM of labelled ADP on the cytoplasmic side and 5 mM of unlabelled ADP on the matrix side so that the influx of labelled ADP can be used as a metric of transport. Fig. 3 shows that the imposition of equal cytoplasmic and matrix network energies, in the absence of substrate binding energy, effectively impedes transport. As a consequence, the residual transport decreases approximately exponentially with increasing strength of the networks. Thus strong networks will prevent changes from matrix to cytoplasmic conformation in the absence of a substrate binding energy, that is, in the absence of bound substrate.

**Fig. 4:**
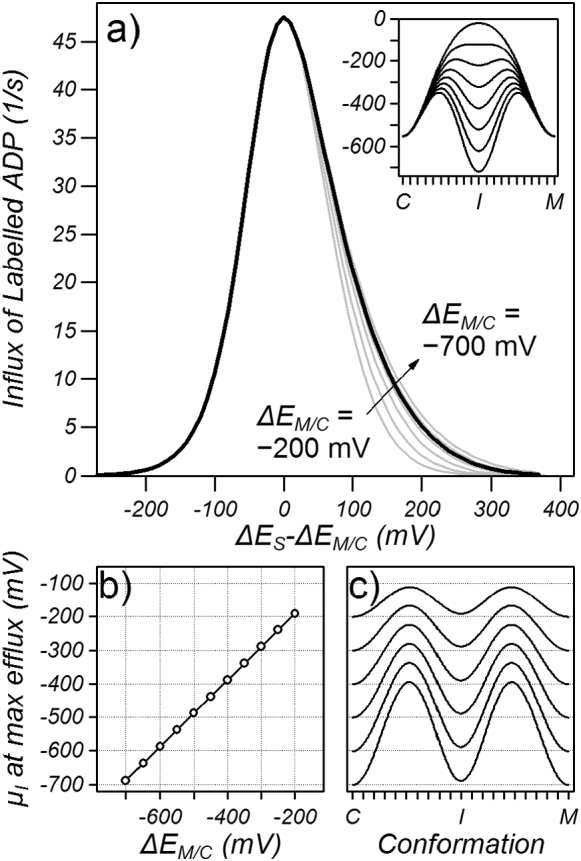
The dependence of transport on the substrate binding energy. a) Transport for different symmetric network strengths (*ΔE*_*M*_ = *ΔE*_*C*_ = *ΔE*_*M/C*_) as a function of substrate binding energy. Simulations were carried out with 5 mM of labelled ADP on the cytoplasmic side and 5 mM ADP on the matrix side and the efflux of ADP plotted as the substrate binding energy was varied at constant symmetric network strength (*ΔE*_*M/C*_). Black line: *ΔE*_*M/C*_ = − 550 mV, grey lines *ΔE*_*M/C*_ = − 200, − 300, − 400, − 500, − 600 and − 700 mV. The insert shows energy profiles for *ΔE*_*M/C*_ = − 550 mV. b) The chemical potential of the intermediate state (μ_I_) at maximum transport rate plotted for a given network strength. C) Energy profiles at maximum transport rate for different *ΔE*_*M/C*_.

The ability of substrate binding to facilitate exchange was examined by running the same simulations with an equal cytoplasmic and matrix network strength of − 550 mV and by varying the substrate binding energy (black line Fig. 4a). It was found that maximum transport occurred when the substrate binding energy was approximately equal to the network strength, as previously proposed [12]. To explore the relationship between the substrate binding energy and network strength further, similar simulations were carried out with a range of equal network strengths from − 200 to − 700 mV (grey lines Fig. 4a) where it was found that maximum transport occurred when the chemical potential of the intermediate state was approximately equal to the cytoplasmic and matrix network strength (Fig. 4b and c). Weak networks lead to faster rates of transport (see Fig. S2 in the supplementary data) but when the rate constant for the conformational change (*k*_*c*_ in Eq. (3)) was set so that the maximum transport rate for each network strength was equal (*k*_*c*_ of 0.065, 0.200, 0.721, 2.810, 11.488 and 48.76/μs for network strengths of − 200, − 300, − 400, − 500, − 600 and − 700 mV, respectively), the relationship between transport rate and substrate binding energy was independent of the absolute network strength, albeit mildly dependent when *ΔE*_*S*_ − *ΔE*_*M/C*_ > 0 (grey lines, Fig. 4a). Importantly, however, the maximum transport rate was strongly dependent on the difference between the substrate binding energy and the network strength (Fig. 4a).

**Fig. 5:**
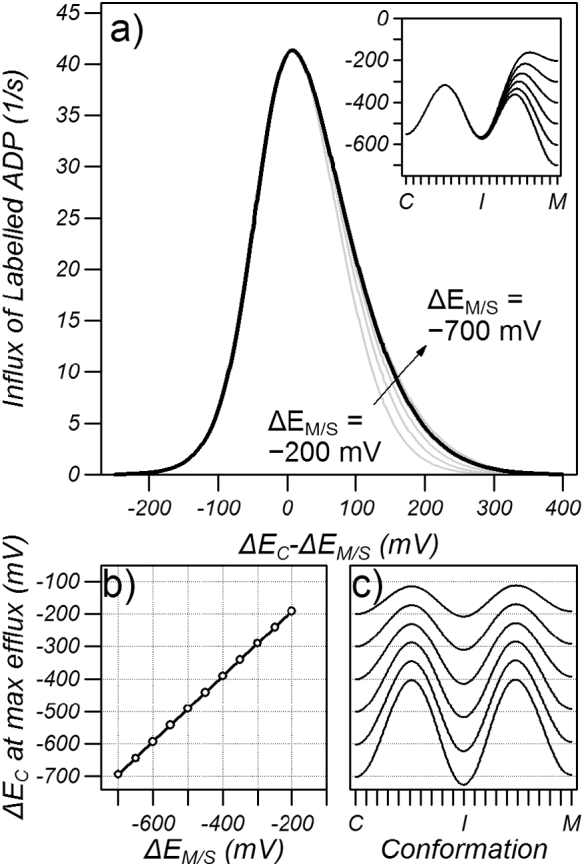
The dependence of transport on the cytoplasmic network strength. a) Transport for different cytoplasmic network strengths (*ΔE*_*C*_) when the matrix network (*ΔE*_*M*_) and substrate binding energy (*ΔE*_*S*_) are equal (*ΔE*_*M/S*_). Simulations were carried out with 5 mM of labelled ADP on the cytoplasmic side and 5 mM ADP on the matrix side and the efflux of ADP plotted as the matrix network strength was varied. Black line: *ΔE*_*M/S*_ = − 550 mV, grey lines *ΔE*_*M/S*_ = − 200, − 300, − 400, − 500, − 600 and − 700 mV. The insert shows energy profiles for *ΔE*_*M/S*_ = − 550 mV. b) The cytoplasmic network strength at maximum transport rate plotted for a given matrix network/substrate binding energy. C) Energy profiles at maximum transport rate for different *ΔE*_*M/S*_.

The dependence of transport on cytoplasmic network strength was determined by running simulations with varying cytoplasmic network strength when the matrix network and the substrate binding energy (*ΔE*_*M/S*_) was set to − 550 mV (Fig. 5a, black line). It was found that maximum transport occurred when the cytoplasmic network strength was very similar to that of the matrix network. Carrying out simulations with a range of matrix network strengths, with equal substrate binding energy (Fig. 5a, grey lines), demonstrated that this property was a general feature of the model and that maximal flux occurred when the strength of the cytoplasmic network was 5–10 mV above that of the matrix network (Fig. 5b). When the rate constant for the conformational change (*k*_*c*_) was varied such that the maximum transport rate was normalized to that of the measured rate, then the dependence on transport rate on relative cytoplasmic network strength was similar (Fig. 5a).

**Fig. 6:**
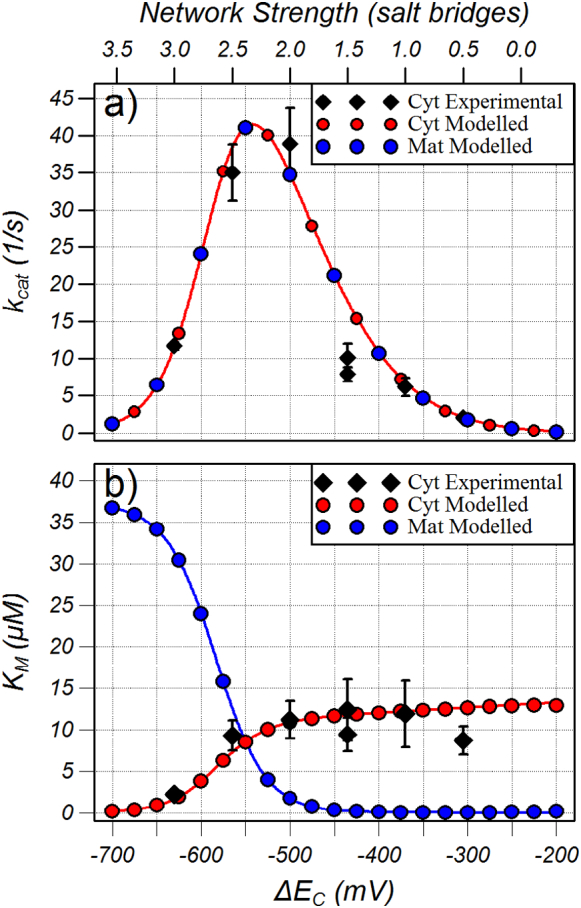
Experimental and modelled *k*_*cat*_ and *K*_*M*_ for transport of cytoplasmic (Cyt) and matrix (Mat) substrate. The modelled points were calculated by performing simulations with *ΔE*_*M*_ and *ΔE*_*S*_ set to − 550 mV with different substrate concentrations in either the cytoplasmic or matrix compartments (with the substrate in the other compartment set to 5 mM), and then fitting the rate of transport to a Michaelis-Menten function. To prevent overlay, only alternate points of cytoplasmic and matrix modelled *k*_*cat*_ were plotted. The experimental values are from the uptake of ^14^C ADP into membrane-fused vesicles when the internal ADP was at 5 mM. The relationship between network strength in salt bridges (experimental data, top x axis) was registered visually with the network strength in millivolts (modelled data, bottom x axis). The lines in a) are from a simplified model described in the supplementary information and the lines in b) from a simplified model described in the Discussion.

The *K*_*M*_ and *k*_*cat*_ for transport from the cytoplasmic and matrix sides were determined by carrying out simulations when the substrate concentration was varied between 0 and 100 μM in 0.1 μM steps with substrate in the other compartment set to 5 mM. The transport of ADP at different substrate concentrations was then fitted to a Michaelis-Menten function using Levenberg-Marquardt non-linear minimization algorithm to determine the *K*_*M*_ and *k*_*cat*_. The results of simulations when the matrix network strength and substrate binding energy were set to − 550 mV are shown in Fig. 6 as a function of the cytoplasmic network strength. As expected, the *k*_*cat*_ for transport was the same for both cytoplasmic and matrix substrate and the maximum transport was found when the cytoplasmic network, the matrix network and the substrate binding energy were all approximately equal (*ΔE*_*C*_ = − 550 mV in Fig. 6a). The *K*_*M*_ for cytoplasmic and matrix substrate were also equal under these conditions (cf. red and blue circles at − 550 mV, Fig. 6b), which would be expected because the model is symmetric about the intermediate conformation when the cytoplasmic and matrix networks have equal strength. This symmetry is broken when the networks have unequal strength and a strong cytoplasmic network resulted in a submicromolar *K*_*M*_ from the cytoplasmic side whereas the *K*_*M*_ from the matrix side approached the *K*_d_ (40 μM). With a weak cytoplasmic network, the *K*_*M*_ from the cytoplasmic side was 12–14 μM, whereas the *K*_*M*_ from the matrix side was submicromolar.

Fig. 6 compares the *k*_*cat*_ and *K*_*M*_ of the model with those determined experimentally using vesicles fused with *L. lactis* membranes expressing wild type or mutant carriers [20]. The carrier protein in the membrane-vesicle fusions was estimated to be 90% orientated with their cytoplasmic side on the outside of the vesicle by their ability to be inhibited by membrane-impermeable carboxyatractyloside, which only inhibits from the cytoplasmic side. The vesicles were loaded with 5 mM ADP and the initial rate, calculated from the linear part of the ^14^C-ADP uptake curve for different external ^14^C-ADP concentrations, was fitted to a Michaelis-Menten function as previously described. The apparent *k*_*cat*_ and *K*_*M*_ values for the experimental cytoplasmic substrate are shown in Fig. 6 as a function of network strength. The experimental and modelled data was registered visually where the cytoplasmic network strength in millivolts was estimated to be ≅ − 130 N − 240 where N is the network interaction energy in number of salt bridges.

**Fig. 7:**
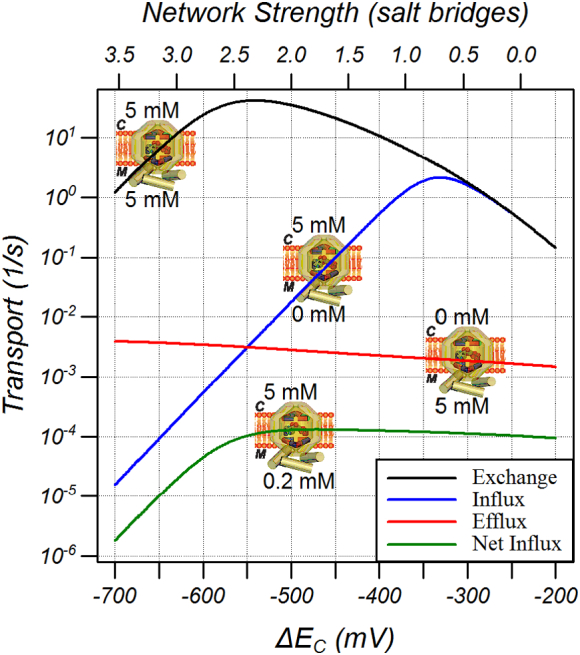
The dependence of exchange and uniport activity on the cytoplasmic network strength. Simulations were carried out with *ΔE*_*M*_ = *ΔE*_*S*_ = − 550 mV and the graphics show the substrate concentration on each side of the membrane.

We have previously suggested that differences in the network strengths would allow the carrier to act as a uniporter rather than an exchanger with the rationale that there would be a higher probability that a weak network would break in the absence of substrate [12]. To test this possibility, simulations were carried out under different substrate gradients when the matrix network strength and substrate binding energy were set to − 550 mV and the strength of cytoplasmic network was varied (Fig. 7). As expected, the carrier acted as an exchanger when 5 mM of labelled and unlabelled substrate was available on opposite sides of the membrane (black line). Very little uniport efflux was observed when substrate was only present on the matrix side, regardless of the cytoplasmic network strength (red line). On the other hand, substantial uniport influx did occur when 5 mM substrate was present only on the cytoplasmic side and when the strength of the cytoplasmic network was weaker than that of the matrix network (blue line). The maximum uniport transport occurred when the cytoplasmic network was ≅ − 330 mV, equivalent to ≈ 0.7 salt bridges when using the comparison introduced in Fig. 6. However, this uniport influx was an order of magnitude lower than the exchange rate when the networks were of equal strength.

Many studies on reconstituted carriers are carried out under idealised conditions with substrate present on only one side of the membrane for uniporters or different substrates present on each side of the membrane for exchange (e.g. ATP and ADP or labelled and unlabelled ADP for the ADP/ATP carrier). This may not be the case for carriers operating under physiological conditions if the downstream enzymes do not further metabolize the substrate sufficiently. In order to test whether returning to the cytoplasmic conformation with or without substrate would affect net uniport activity, simulations were carried out when 5 mM of substrate was present on the cytoplasmic side and 0.2 mM of substrate was present on the matrix side. Under these conditions the carrier both exported and imported substrate. It was found that net substrate influx, which is the uniport component of transport, was almost abolished (Fig. 7, green line), although the carrier exchanged substrates at high rates determined by the *K*_*M*_ on the matrix side. This result indicates that a carrier with a weak cytoplasmic network would not function well as a uniporter under conditions where substrate was present on both sides of the membrane.

## 4 Discussion

Our model was able to reproduce the dependency of both *k*_*cat*_ and *K*_*M*_ on the cytoplasmic salt bridge network strength (Fig. 6) with good quantitative accuracy and with very few free parameters implying that the model captures the essence of the transport mechanism and provides a framework to understand the structure-function relationships of this class of transporters. The model confirms that the cytoplasmic and matrix networks not only close access of the binding site to the respective compartments but also provide the free energy barrier that prevents conformational changes in the absence of substrate.

In the steady state, the transport rate, *J*_*T*_, is equal to the net flux of a substrate through any of neighbouring confirmations, *J*_*n*_^*c* , *s* →* c* + 1 , *s*^, and is given by:

(7) $$\begin{array}{lll} J_{T} & = & J_{n}^{c,s\to c+1,s}\\ & = & J^{c,s\to c+1,s}-J^{c+1,s\to c,s}\\ & = & P_{c,s}k^{c,s\to c+1,s}-P_{c+1,s}k^{c+1,s\to c,s} \end{array}$$

where *J*^*c* , *s* →* c* + 1 , *s*^ and *J*^*c* + 1 , *s* →* c* , *s*^ are the forward and reverse fluxes, *P*_*c* , *s*_ and *P*_*c* + 1 , *s*_ are the probability that the carrier is found in the neighbouring conformations *c* and *c* + *1*, respectively, with substrate *s* bound, and *k*^*c* , *s* →* c* + 1 , *s*^ and *k*^*c* + 1 , *s* →* c* , *s*^ are the forward and reverse rate constants, respectively, for the conformational change. In our model of the carrier as a nanomachine, transport is not limited by the rate constants, which are five orders of magnitude greater than the *k*_*cat*_ of transport. Instead, it is the very low probability of attaining the highest energy states that limit the forward and reverse fluxes, and hence the net flux.

In the absence of a substrate binding energy (conditions of Fig. 3a), the probability distribution of the carrier conformation will reach thermal equilibrium in the steady state and be governed by Boltzmann's distribution (see S.1 in the Supplementary information for a proof) such that the probability *P*_*c*_ that the carrier is in conformation c will be given by:

(8) $$P_{c}=\frac{e^{-\mu_{c}^{0}/{k_{B}}^{T}}}{e^{-\mu_{-10}^{0}/{k_{B}}^{T}}+e^{-\mu_{-9}^{0}/{k_{B}}^{T}}+\cdots +e^{-\mu_{0}^{0}/{k_{B}}^{T}}+\cdots +e^{-\mu_{+9}^{0}/{k_{B}}^{T}}+e^{-\mu_{+10}^{0}/{k_{B}}^{T}}}$$

where the denominator is the partition function. The forward flux is then given by *J*_*f*_^*c* , *s* →* c* + 1 , *s*^ = *F*_*c* , *s*_*P*_*c*_*k*_*f*_^*c* , *s* →* c* + 1 , *s*^ where *F*_*c* , *s*_ is the fraction of carriers in conformation *c* that have substrate *s* bound. While the conformation is in close thermal equilibrium, the states of the model are not in equilibrium because there is a gradient in the fraction of substrate bound at different conformations (Fig. S.1 of the Supplementary data) and this gradient allows net transport to occur.

The conformation with the smallest forward flux is the intermediate conformation (*c* = *0*) because it has the highest chemical potential and hence the numerator of Eq. (8) is smallest. The denominator is dominated by the matrix and cytoplasmic conformations (most left and most right terms) because they have the lowest chemical potential. The probability of the carrier being in the intermediate state *P*_*I*_ is approximated by *P*_*I*_ ≈ ½*e*^−(*μ*_*I*_^0^ − *μ*_*N*_^0^)/*k*_*B*_*T*^ where *μ*_*I*_^0^ and *μ*_*N*_^0^ are the standard chemical potentials of the intermediate and cytoplasmic/matrix conformation, respectively, and the factor ½ accounts for both the cytoplasmic and matrix terms appearing in the denominator of Eq. (8). Thus the difference in chemical potential between the cytoplasmic/matrix conformation and the intermediate conformation represents an energy barrier that results in a low probability of the carrier reaching the intermediate conformation. This analysis is confirmed when the forward flux, calculated with Eq. (8) and assuming the fraction of bound labelled ADP is 0.5, is compared to the transport rates (Fig. 3) where it is found that the forward flux is only 60% larger than the net flux at high network strengths. This simple analysis of barrier height dictating transport rate is verified when the net flux is estimated (see S.4 in the Supplementary data), whereupon the barrier is dependent on the mean chemical potential of the highest energy neighbouring conformations rather than simply the highest energy conformation.

The previous analysis is still a good approximation if a substrate binding energy is non-zero as long as the concentration of substrate on both sides of the carrier is high, ensuring the probability of unbound carrier is near zero. In this case, the energy levels refer to the carrier with bound substrate.

**Fig. 8:**
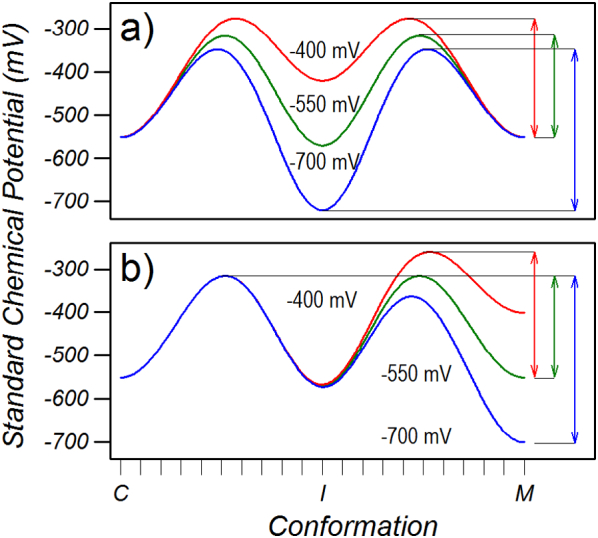
Energy profiles and transport energy barriers shown as arrows between the lowest energy state(s) and the highest energy state(s). a) symmetric network strength of − 550 mV with substrate binding energies of − 400, − 550 and − 700 mV. b) A matrix and substrate binding energy of − 550 mV with a cytoplasmic network of − 400, − 550 and − 700 mV.

With the imposition of a substrate binding energy, the conformation with minimum probability shifts from the intermediate conformation to the two maxima with equal chemical potential between the intermediate conformation and the cytoplasmic and matrix conformations (Fig. 8a, red trace). Under these conditions the energy profile has two maxima and two minima. Similar to the presence of two minimum energy states (cytoplasmic and matrix) doubling the denominator of Eq. (8) and halving the transport rate, the effect of two equal maxima is also to halve the transport rate compared to one maximum (see Supplementary data S.5). Furthermore, a rigorous analysis shows that the maximum of the energy barrier is an average of all the states exponentially weighted to the high energy states and that the minimum of the energy barrier is also an average but exponentially weighted to the low energy states (Supplementary data S.5). Because the weighting is exponential, the barrier maximum and minimum is dominated by the high and low energy states, respectively, the energy barrier can be estimated simply from the difference between the maximum and minimum of the energy profile (Fig. 8a, red arrow). Increasing the substrate binding energy initially reduces the energy barrier (Fig. 8a green arrow) as the maximum is decreased until the standard chemical potential of the intermediate state falls below the cytoplasmic and matrix conformations whereupon the energy barrier, which is now between the intermediate state and the two maxima, increases with increasing substrate binding energy (Fig. 8a, blue arrow). The transport rate therefore increases with increasing substrate binding energy up to a maximum when the chemical potential of the intermediate conformation is approximately equal to that of the cytoplasmic and matrix conformation, whereupon the energy barrier is at a minimum, and then decreases with further increases in substrate binding energy. This analysis shows that the energy barrier is not an activation energy, which would be defined as the difference between the cytoplasmic and intermediate conformations and would determine the rate constant for the conformational change between the cytoplasmic and intermediate conformation, but is rather the difference between energy maximum and energy minimum that sets the probability of being in the high energy conformation and hence determines the flux.

Similarly, when the cytoplasmic network is weaker than the matrix network, the matrix maximum rises in energy and the energy barrier, which is between the cytoplasmic conformation and the matrix maximum, increases (Fig. 8b red trace, red arrow), decreasing the transport rate (Fig. 5a). In contrast, when the cytoplasmic network is stronger than the matrix network, the chemical potential of the matrix maximum and matrix conformation is lowered such that the energy barrier, which is now between the cytoplasmic maximum and matrix conformation, increases (Fig. 8b blue trace, blue arrow), decreasing the transport rate. The minimum energy barrier occurs when the cytoplasmic network is equal in strength to the matrix network and the substrate binding energy, whereupon the transport rate is a maximum. The similarity in the dependence of energy barrier on substrate binding energy and on the cytoplasmic network strength (Fig. 8a and b, respectively) explains the similarity of the dependence of transport on substrate binding energy and on the cytoplasmic network strength (Figs. 4a and 5a, respectively).

**Fig. 9:**
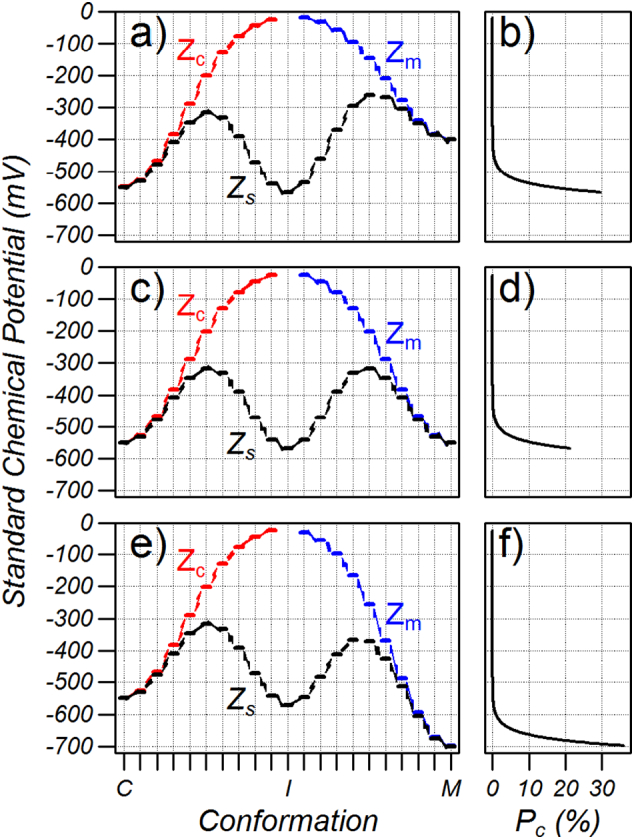
The partition function for the carrier with *ΔE*_*M*_ = *ΔE*_S_ = − 550 mV and *ΔE*_*C*_ = − 400 mV, − 550 and − 700 mV for the upper middle and lower graphs, respectively. a), c) and e) States contributing to the unbound cytoplasmic (Z_c_), unbound matrix (Z_m_) and substrate bound (Z_s_) partial partition functions. b), d) and f) Normalized Boltzmann coefficients (*P*_*c*_).

At non-saturating substrate concentrations, the probability of the carrier without substrate bound becomes significant and these states must be included in the partition function. The rate of substrate binding and release is much faster than transport [23] so that the unbound cytoplasmic and matrix conformation will be maintained in close equilibrium with the corresponding substrate-bound conformation. With these approximations, Eq. (8) can be rewritten

(9) $$P_{c}=\frac{e^{-\mu_{c}^{0}/{k_{B}}^{T}}}{\frac{K_{d}}{\left[{\mathrm{ADP}}_{c}\right]}Z_{c}+Z_{s}+\frac{K_{d}}{\left[{\mathrm{ADP}}_{m}\right]}Z_{m}}$$

where the denominator is the partition function, [ADP_c_] and [ADP_m_] are the concentrations of ADP on the cytoplasmic and matrix side, respectively, *Z*_*s*_ are the terms of the partition function of the substrate-bound carrier shown in the denominator of Eq. (8), and *Z*_*c*_ and *Z*_*m*_ are the terms of the partition function for the unbound carrier on the cytoplasmic and matrix side, respectively (see Fig. 9). The factors *K*_*d*_/[*ADP*_*c*_] and *K*_*d*_/[*ADP*_*m*_] account for the *ΔE*_*B*_ of substrate binding such that *Z*_*c*_ and *Z*_*m*_ are independent of substrate concentration (see S.3 of the Supplementary information). Note that Eq. (9) tends to Eq. (8) when the substrate concentrations are very large. From this, the *K*_*M*_ for transport on the cytoplasmic and matrix side, *K*_*M*_^*c*^ and *K*_*M*_^*m*^, respectively, are given by:

(10) $$\begin{array}{l} K_{M}^{c}=\frac{Z_{c}}{Z_{s}}K_{d}\\ K_{M}^{m}=\frac{Z_{m}}{Z_{s}}K_{d} \end{array}$$

and plotted as the lines in Fig. 5 showing an excellent agreement between this approximation and the full model.

When the cytoplasmic and matrix networks are equal in strength then *Z*_*c*_ = *Z*_*m*_ and the *K*_*M*_'s are equal. Weakening the cytoplasmic network (Fig. 9a and b) decreases *Z*_*s*_ by a maximum factor of 2 as the right hand terms of the denominator vanish. *Z*_*c*_ is independent of the cytoplasmic network strength so the *K*_*M*_ on the cytoplasmic side increases by a maximum of a factor of 2. *Z*_*m*_ strongly decreases with weakening of the cytoplasmic network and hence the *K*_*M*_ on the matrix side decreases to near zero. Strengthening the cytoplasmic network strongly increases *Z*_*s*_ and *Z*_*m*_ while decreasing *Z*_*c*_ and the *K*_*M*_ on the cytoplasmic side decreases to near zero.

The model clearly predicts that the maximum transport occurs when the interaction energies of the cytoplasmic network, matrix network and substrate binding are approximately equal such that the energy barrier is a minimum. The wild type carrier has a matrix network of three salt bridges braced by a hydrogen bond from a glutamine residue [9]. In the semi-quantitative measure introduced before in which a hydrogen bond has half the strength of a salt bridge, this extra interaction would give the matrix network a total strength of 3.5 salt bridges. The phosphate groups of ADP may form salt bridges with the three positively charged residues of the binding site and the adenine ring may form a π-stacking arrangement with a tyrosine residue [5], [10], [12], [14], [15] thus giving the substrate binding energy a total strength of 3.5, similar to that of the matrix network. This equal strength between the network and the substrate binding energy is predicted by the model to give maximum *k*_*cat*_. The cytoplasmic network of the fungal ADP/ATP carrier consists of two salt bridges and a hydrogen bond giving it a total strength of 2.5. It would therefore be predicted that mutating the hydrogen bond of the cytoplasmic network to a salt bridge would increase the *k*_*cat*_ compared to the wild type. Unexpectedly, the *k*_*cat*_ of this mutant is actually decreased and the carrier with the greatest *k*_*cat*_ is the mutant with a cytoplasmic network strength 0.5 salt bridges weaker (Fig. 6 and Table S.1) indicating that the combined interaction energy of the wild type carrier in the matrix conformation must be approximately half a salt bridge greater than in the cytoplasmic conformation. Further experimental evidence for this is that the *K*_*M*_ for transport is larger from the matrix (25 μM) than the cytosol (10 μM) [29], as would be predicted by the model for a stronger cytoplasmic network (Fig. 6). Simply counting the number of salt bridges and hydrogen bonds is a coarse form of quantitation and the free energy of formation of the bond will depend on the bond length and unfavourable desolvation that must occur to form a salt bridge [30] but this work predicts that there must be additional interactions involved in the stabilisation of the matrix conformation with a strength equivalent to 1.5 salt bridge interactions. There is a highly conserved symmetrical motif [*FW*]xx[YF] present at the cytoplasmic side of the carrier [9], [31], [32]. These aromatic residues could be involved in multiple aromatic stacking arrangements, each approximately equivalent to a hydrogen bond, which could increase the overall interaction energy of the matrix conformation. Confirmation of this hypothesis will have to await a crystal structure of the carrier in the matrix conformation.

A recent paper has attempted to map out the free energy landscape of the carrier conformation change using well-tempered metadynamics [33]. The authors have calculated that the cytoplasmic and matrix conformations are separated by an energy barrier of ≈ 430 mV (10 kcal/mol) in the absence of substrate, which would robustly prevent leak (Fig. 1). Our analysis would predict that maximum transport would occur with a *ΔE*_*S*_ of ≈ 400 mV which would lower the energy barrier by ≈ 230 mV. Binding of ADP generated a minimum in the free energy landscape ≈ 260 mV below the energy barrier in the absence of substrate [33], but this minimum was offset from the conformation with maximum energy and only lowered the energy barrier by ≈ 86 mV. Our interpretation of the modelling concludes that it is the lowering of the energy barrier, and not the presence of an energy minimum, that allows substrate transport. Lowering the barrier by only 86 mV would not produce rapid transport compared to the 230 mV predicted by our model for maximum transport (Fig. 4c). Furthermore, binding of ATP did not generate a significant energy minimum in the intermediate conformation compared to the empty carrier, or lower the energy barrier, suggesting that the binding energy of ATP did not increase in the intermediate conformations. The failure of substrate to lower the energy barrier substantially in the metadynamics simulations could be caused by carrying out insufficient iterations for the simulation to fully probe state space and find those states in which the substrate is tightly bound before the applied potential drives the conformational changes further. Moreover, the structures in the simulations are not consistent with an alternating access mechanism, as there is no substrate-occluded intermediate state or a closed matrix state involving the formation of the cytoplasmic network, as experimentally shown [9], [20].

The mitochondrial carrier family, in which the matrix and cytoplasmic networks are common motifs, include both exchangers and uniporters. The modelling shows that very unequal network strengths can lead to unidirectional uniport activity in the direction from weak to strong network (Fig. 7), because the energy barrier for breaking a weak network in the absence of substrate is sufficiently small that the carrier can return to the conformation with the strong network engaged without bound substrate. However, the cost of reducing the return energy barrier is that the forward energy barrier is larger, even in the presence of substrate (Fig. 8a), and hence transport rates are slower. Furthermore, the energy barrier for return is always smaller when substrate is bound than when not bound such that return with substrate is preferred and there is negligible uniport activity when substrate is present on both sides of the membrane (Fig. 7). For a mitochondrial carrier that imports substrate, such as the glutamate carrier that imports glutamate into the matrix where glutamate dehydrogenase is exclusively located [34], or the inorganic phosphate carrier that imports phosphate into the matrix for conversion with ADP to ATP by the ATP synthase, this would require a weak cytoplasmic network and a low concentration of substrate in the matrix. Both transporters carry out substrate transport in symport with a proton (or antiport with a hydroxyl ion) such that the net transport is electroneutral. The substrate binding site of the phosphate carrier contains positively charged residues, which could form salt bridges to the phosphate, and a glutamate residue that could aid proton binding for symport [12]. Likewise, the glutamate carrier also contains a glutamate in the binding site suggesting it too symports a proton [12]. Symport would require that the two substrates bind co-operatively, that is, the *K*_d_ of binding one substrate is much lower when the other substrate is already bound, so that the probability of only having one substrate bound is very low. The glutamate carrier has a matrix network of three salt bridges and a cytoplasmic network lacking any salt bridges or hydrogen bonds [12] with a very low *k*_*cat*_ for homo exchange (0.083 and 0.019 s^− 1^ for isoform 1 and 2, respectively [34]) suggesting that it achieves uniport activity by unequal network strengths, consistent with the findings of our modelling. The uniport activity is 10–30% of the homo exchange [34] but the net uniport activity in the presence of internal glutamate has not been measured. In contrast, the phosphate carrier has equal mitochondrial and cytoplasmic networks, consisting of two salt bridges [12] and has higher activity than the ADP/ATP carrier [29]. The relatively weaker network strength would explain the higher activity for homo-exchange compared to the ADP/ATP carrier but the uniport activity should be vanishingly small whereas it has been measured to be similar to exchange activity [35]. Furthermore, the phosphate uniport action is bidirectional (uniport can occur in both directions) [36] whereas a strong/weak network combination would be expected to generate only unidirectional transport. Together, this would suggest that the phosphate carrier employs an alternative mechanism to substitute for the substrate binding energy during the change between conformations when substrate is not bound.

## Transparency document

Transparency document Image 1

## Appendix A Supplementary data

Supplementary material Image 2
