# Supplementary material for: Modelling the free energy profile of the mitochondrial ADP/ATP carrier
Source: Biochim Biophys Acta. 2017 Nov;1858(11):906–14. doi: 10.1016/j.bbabio.2017.05.006 (PMC5604490; doi:10.1016/j.bbabio.2017.05.006)
Supplement: Supplementary file 2 — Supplementary material [file mmc2.pdf]

Supplementary Data

S.1 Transport parameters of the mutants

Mutant	Network strength	Apparent K_M (μM)	k_{cat} (1/s)	Initial Rate (1/s)
Q302K	3.0	2.20±0.30	11.68±0.48	3.31±0.17
Wild type	2.5	9.30±1.80	35.04±3.79	5.72±0.43
Q302A	2.0	11.20±2.30	38.93±4.80	4.84±0.52
K104A	1.5	12.40±3.70	10.13±1.87	0.84±0.05
K208A	1.5	9.40±2.00	7.89±0.96	0.79±0.18
K104A+Q302A	1.0	11.90±4.00	6.19±1.23	0.46±0.11
K104A+K208A	0.5	8.70±1.70	2.08±0.27	0.49±0.05

Values are mean±SD (n=4). k_{cat} and initial rate were calculated from specific activities using a molecular weight of 32 kDa. Values are for external substrate when the internal concentration of ADP was at 5 mM. The initial rate is the initial rate of accumulation of 1.5 μM of external ^{14}C-ADP. Data were originally published in [21].

S.2 Thermal Distribution of the Carrier in the Absence of ΔE_S

The net flux of the conformational change between conformations *c* and *c+1* is the sum of the net fluxes of the empty carrier and that with substrate bound, e .g.

$$J_n^{c\to c+1} = \sum_s \left(J_n^{c,s\to c+1,s}\right) = \sum_s \left(P_{c,s} k^{c,s\to c+1,s} - P_{c+1,s} k^{c+1,s\to c,s}\right) \quad \text{Equation s1}$$

Where the sum is over the s substrates which are either none, ADP or labelled ADP, and $P_{c,s}$ is the probability of the carrier being in conformation c with substrate s bound. In the absence of a substrate binding energy, the forward and reverse rates are independent of the substrate bound such that:

$$J_n^{c\to c+1} = k^{c\to c+1} \sum_s P_{c,s} - k^{c+1\to c} \sum_s P_{c+1,s} \quad \text{Equation s2}$$

Where $k^{c\to c+1}$ and $k^{c+1\to c}$ are the forward and reverse rates for the change between conformations *c* and *c+1*. The first sum in equation s2 is the sum of the probability of the carrier being in the cth conformation with the substrate binding site being empty or bound with ADP which is simply equal to the probability of the carrier being in the cth conformation (P_c). Likewise for the second sum, but in conformation *c+1*. Furthermore, the net flux from conformation *c* to *c+1* must be zero in the steady state, hence equation s2 can be expressed as

$$0 = P_c k^{c\to c+1} - P_{c+1} k^{c+1\to c} \quad \text{Equation s3}$$

Which can be rewritten, using equation 1 from the manuscript, as:

	$P_{c+1} = P_c \frac{k^{c\to c+1}}{k^{c+1\to c}} = P_c e^{\left(\mu_c^0 - \mu_{c+1}^0\right)/K_bT}$	Equation s4

which defines the Boltzmann distribution. To be more explicit, equation s4 can be expressed as

	$P_i e^{-\mu_c^0/k_BT} = P_c e^{-\mu_i^0/k_BT}$	Equation s5

where *c* and *i* are any conformation. The probabilities of being in each conformation must sum to 1, e.g. $\sum_i P_i = 1$, thus

	$\sum_i P_i e^{-\mu_c^0/k_BT} = e^{-\mu_c^0/k_BT}$	Equation s6

Using equation s5, equation s6 can be written as the standard form of Boltzmann's distribution, e.g.

	$P_c = \frac{e^{-\mu_c^0/k_BT}}{\sum_i e^{-\mu_i^0/k_BT}}$	Equation s7

Where the denominator is the partition function, Z.

S.3 Thermal Distribution of the Carrier in the Presence of ΔE_S

Whereas the previous analysis is exact, a similar analysis can be carried out in the presence of a substrate binding energy if it is assumed that (1) the net flux through the unbound conformations is zero and (2), that the rapid binding of substrates compared to transport ensures good equilibrium between the bound and unbound cytoplasmic and matrix conformations. The zero flux through the unbound conformations will then ensure that unbound conformations on the cytoplasmic side of the intermediate conformation will be in equilibrium with the cytoplasmic conformation and likewise for the matrix conformations.

The partition function will be the sum over all the states and given by

	$Z = \sum_{c=-10}^{1} e^{-\mu_0^{c,e}/k_BT} + \sum_{c=-10}^{10} e^{-\mu_0^{c,s}/k_BT} + \sum_{c=1}^{10} e^{-\mu_0^{c,e}/k_BT}$	Equation s8

Where the first sum is over the unbound states of the cytoplasmic side, the central term is over the substrate bound states and the last term is over the unbound states on the matrix side. If the zero of figure 2 is offset by ΔE_B, then the chemical potentials of the bound ($\mu_0^{c,s}$) and unbound ($\mu_0^{c,e}$) states are given by:

	$\mu_0^{c,e} = \Delta E_M^c + \Delta E_C^c - \Delta E_B$<br>$\mu_0^{c,s} = \Delta E_M^c + \Delta E_C^c + \Delta E_S^c$	Equation s9

Where ΔE_B is the free energy change of substrate binding given by equation 6. Thus equation 5 can be written

	$Z = \frac{K_d^s}{[Sc]} Z_c + Z_s + \frac{K_d^s}{[Sm]} Z_m$	Equation s10

Where [*Sc*] and [*Sm*] are the concentration of substrate on the cytoplasmic and matrix side, respectively, Z_c, Z_s and Z_m are partial partition functions representing the sums of the unbound cytoplasmic state, the substrate-bound states and the unbound matrix states, respectively and given by

	$Z_c = \sum_{c=-10}^{1} e^{-\left(\Delta E_M^c + \Delta E_C^c\right)/k_B T}$<br>$Z_s = \sum_{c=-10}^{10} e^{-\left(\Delta E_M^c + \Delta E_C^c + \Delta E_S^{c,s}\right)/k_B T}$<br>$Z_m = \sum_{c=1}^{10} e^{-\left(\Delta E_M^c + \Delta E_C^c\right)/k_B T}$	Equation s11

S.4 Estimation of the Transport Rate

The transport rate J_T is equal the net flux of ADP between the *c+1* and *c* conformation and given by

	$J_T = J_n^{c+1,s \to c,s} = F_{c+1,s} P_{c+1} k^{c+1,s \to c,s} - F_{c,s} P_c k^{c,s \to c+1,s}$	Equation s12

Where *s* is ADP and $F_{c,s}$ is ADP fraction of bound substrate. Using equation s3 ($P_c k^{c,s \to c+1,s} = P_{c+1} k^{c+1,s \to c,s}$), this can be rewritten

	$F_{c+1,s} = F_{c,s} + \frac{J_T}{P_{c+1} k^{c+1,s \to c,s}}$	Equation s13

Using the formulation of the reverse rate in terms of the chemical potential of the carrier (equation 3) and the probability of the carrier being in conformation c (equation s7), equation s13 can be rewritten:

	$F_{c+1,s} = F_{c,s} + \frac{Z J_T}{k_c e^{-½\left(\mu_{c+1,s}^0 + \mu_{c,s}^0\right)/k_B T}}$	Equation s14

Repeated application of equation s14 can be used to calculate the fraction of ADP bound between the matrix and cytoplasmic conformations as:

	$F_{10,s} = F_{-10,s} + \frac{Z J_T}{k_c} \sum_{c=-10}^{9} e^{½\left(\mu_{c+1,s}^0 + \mu_{c,s}^0\right)/k_B T}$	Equation s15

When only ADP is present in the matrix then $F_{10,s}=1$ and when only labelled ADP is present in the cytosol the $F_{-10,s}=0$ so that the transport rate can be calculated as:

	$J_T = \frac{k_c}{Z} \left(\sum_{c=-10}^{9} e^{½\left(\mu_{c+1,s}^0 + \mu_{c,s}^0\right)/k_B T} \right)^{-1}$	Equation s16

This estimation of the transport rate under conditions of saturating substrate is plotted as the line in figure 6a and compared to the k_{cat} calculated from the full model showing excellent agreement confirming that the estimations made are applicable.

The largest term in the sum of equation s16 is the term in which the neighbouring conformations have the highest chemical potential. Assuming that this term dominates, equation s16 is similar to the estimation of the forward flux in the Discussion except that the numerator is $e^{-½\left(\mu^0_{c+1,s}+\mu^0_{c,s}\right)/k_BT}$, which is the average chemical potential of the highest energy neighbouring states.

S.5 The Energy Barrier of Transport

Most of the free energy profiles show two energy maxima rather than one because the substrate binding energy has created a minimum in the intermediate conformation. When there are two maximums in the free energy profile, these maximums will dominate equation s16 which can be approximated as:

	$J_T = \frac{k_c}{Z}\left(\frac{1}{e^{\mu^0_1/k_BT}+e^{\mu^0_2/k_BT}}\right)$	Equation s17

where μ^0_1 and μ^0_2 are the average chemical potentials of the highest neighbouring states. If the two maximums are equal, then energy barrier is from the lowest energy state (partition function term) to the highest state but the transport is half that of only one barrier. If one maximum is greater than the other, then the larger one quickly dominates then denominator in equation s16 and the transport occurs as if there were only one barrier which is from the lowest to highest energy states.

A formal definition of the energy barrier can be made if equation s17 is written out in full as

	$J_T = k_c\frac{\left(e^{\mu^0_{-9.5}/k_BT}+e^{\mu^0_{-8.5}/k_BT}+\cdots+e^{\mu^0_{+8.5}/k_BT}+e^{\mu^0_{+9.5}/k_BT}\right)^{-1}}{e^{-\mu^0_{-10}/k_BT}+e^{-\mu^0_{-9}/k_BT}+\cdots+e^{-\mu^0_{0}/k_BT}+\cdots+e^{-\mu^0_{+9}/k_BT}+e^{-\mu^0_{+10}/k_BT}}$	Equation 18

where $\mu^0_{-9.5}$ represents the mean of μ^0_{-10} and μ^0_{-9}. If the maximum and minimum of the barrier, E_{max} and E_{min} respectively, are defined as

	$E_{\max} = +k_bT\,\mathrm{Ln}\left(e^{+\mu^0_{-9.5}/k_BT}+e^{+\mu^0_{-8.5}/k_BT}+\cdots+e^{+\mu^0_{+8.5}/k_BT}+e^{+\mu^0_{+9.5}/k_BT}\right)$<br>$E_{\min} = -k_bT\,\mathrm{Ln}\left(e^{-\mu^0_{-10}/k_BT}+e^{-\mu^0_{-9}/k_BT}+\cdots+e^{-\mu^0_{+9}/k_BT}+e^{-\mu^0_{+10}/k_BT}\right)$	Equation 19

whereupon the highest energy states will dominate E_{max} and the lowest energy states will dominate E_{min}. Equation 18 can be rewritten as

	$J_T = k_c e^{-(E_{\max}-E_{\min})/k_bT}$	Equation 20

which relates transport to the energy barrier, which is defined as the difference bewteen E_{max} and E_{min}.

S.6 Transport and the Gradient of Bound Substrate

The fraction of ADP bound to the carrier at each conformation can be calculated from equations s15 and s16 as:

	$F_{n,s} = \dfrac{\sum_{c=-10}^{n-1} e^{½\left(\mu^0_{c+1,s}+\mu^0_{c,s}\right)/k_B T}}{\sum_{c=-10}^{9} e^{½\left(\mu^0_{c+1,s}+\mu^0_{c,s}\right)/k_B T}}$	Equation s21

Figure s1 shows the calculated fraction of bound substrate as a function of carrier conformation using the full kinetic model. The simulations set the cytosolic and matrix concentrations of unlabeled ADP and labelled ADP (ADP*), respectively, to 5 mM to ensure substrate was saturating and the matrix network and substrate binding energies were set to −550 mV with the cytoplasmic network strength set to −400, −550 and −700 mV in figures a), b) and c), respectively.

As predicted, the bound substrate in the cytoplasmic and matrix conformations equilibrate with concentrations of substrate in the respective compartments so that 100% of the carrier in the cytoplasmic conformation has unlabelled ADP bound, 0% labelled, with the opposite true in the matrix conformation.

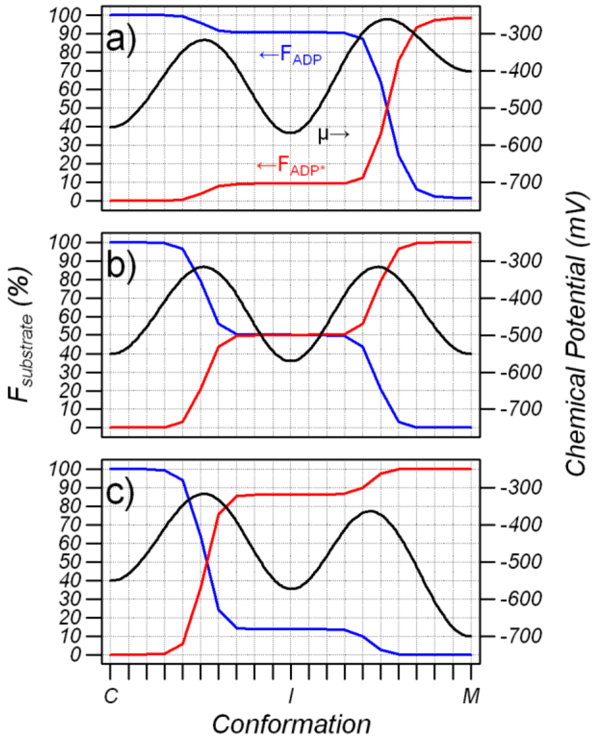

Figure s1. The fraction of bound labelled and unlabelled ADP in each conformation during transport for different cytoplasmic network strengths. Simulations were carried out with 5mM of ADP on the cytoplasmic side, 5mM of labelled ADP (ADP*) on the matrix side, ΔEm and ΔEs of −550mVand with ΔE_c of −400 (a), −550 (b) and −700mV (c). The black line is the chemical potential of the substrate bound carrier, arrows indicate corresponding y-axis.

The transport rate is given by equation s12. Because the carrier operates at thermal equilibrium the terms $P_c k^{c,s\rightarrow c+1,s}$ and $P_{c+1} k^{c+1,s\rightarrow c,s}$ are equal (equation s3), that is, the forward flux for change between conformations *c* and *c+1* is equal to the reverse flux. The transport rate can then be written as:

	$J_T = \left(F_{c+1,s} - F_{c,s}\right) J_i^{c,s\rightarrow c+1,s}$	Equation s22

Where $J_i^{c,s\rightarrow c+1,s}$ is the instantaneous flux equal to the forward and reverse flux.

For low energy conformations the instantaneous flux is very high because the probability of being in the conformation is high hence the fraction of bound substrate in neighbouring conformations can be very similar but still maintain the transport rate. Conversely, high energy states have a low instantaneous flux because the probability of being in the conformation is low and the change in the fraction of bound substrate must be very high to support the transport rate. This is borne out in figure s1, which shows that the gradient in the fraction of bound substrate with respect to the conformation follows the chemical potential of the carrier.

S.7 Transport rates and substrate binding energy.

Figures S2 shows transport rates as a function of substrate binding energy for models with equal cytoplasmic and matrix network strengths. Each line represents transport for different network strengths given in the legend. These simulations were different from those in figure 4a in that the conformational rate constant (k_c in equation 3) was set the same in all the models, whereas in figure 4a, k_c was adjusted in each model to set the maximum transport rate to be the same in all models. This supplementary data is given to show that the maximum transport decreases approximately

exponentially with increasing network strength. Our model cannot be used to estimate the absolute strength of the networks because, although the dependency of the turnover on cytoplasmic network strength depends strongly on the difference between matrix and cytoplasmic network strength, the dependence on the absolute network strength is very small (figure 4).

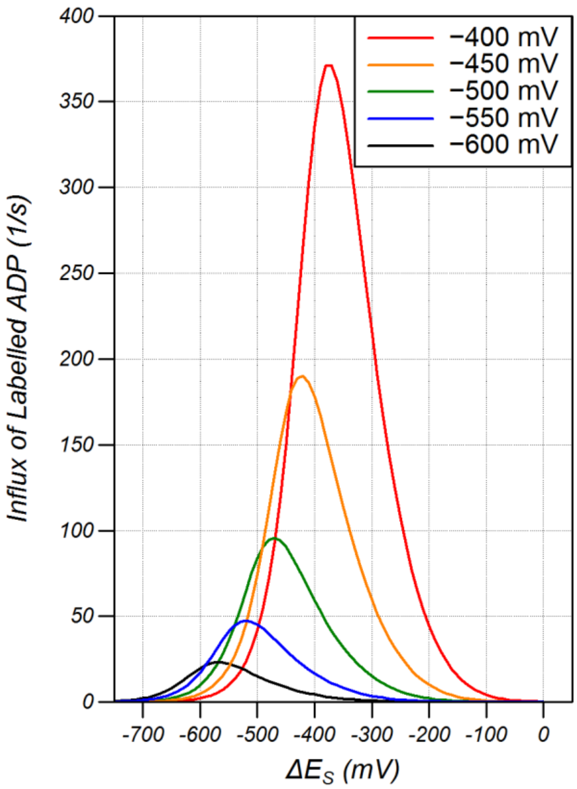

Figure S2 Transport rates as a function of substrate binding energy ΔE_S for different equal cytoplasmic and matrix network strengths, as given in the legend.

S.8 Thermodynamic analysis of the model

Consider a system of a large number of transporters, *N*, embedded in a membrane which separates the system into two compartments (e.g. matrix and cytosolic) with chemical species, *c*, treated separately in the two compartments. The Gibbs free energy of a system, measured in Joules, is defined as

$$G = U + pV - TS + \sum_c n_c \mu_c \qquad \text{Equation s23}$$

Where *U* is the internal energy of the transporters, *p* is the pressure *V* is the volume of the transporter, *T* is the temperature in Kelvin, *S* is the entropy, n_c is the quantity of chemical species *c* in moles and μ_c is the chemical potential of species *c*. This assumes the internal energy and entropy of the other components (membrane and solvent) are constant. The chemical potential of chemical species *c* can be calculated from

$$\mu_c = \mu_c^0 + RT\,\mathrm{Ln}([c]) \qquad \text{Equation s24}$$

where μ_c^0 is the chemical potential under standard conditions, R is the gas constant and [*c*] is the activity of *c* defined as the concentration of *c* divided by the concentration of *c* under standard conditions; [*c*] is equal to 1 under standard conditions.

In a constant pressure system, the term *pV* represents the work done against the pressure due to the volume of the transporter. For simplicity we assume that the volume of the transporter does not change so that the term *pV* can be ignored. If the volume does change, then this term can be included in the internal energy. The transporters can be in different states, *s*, where N_s is the number

of transporters in state *s* such that the total number of transporters is *N*. The entropy of the transporters is given by:

	$S = -k_B \sum_s N_s \, \mathrm{Ln}\left(N_s / N\right)$	Equation s25

where k_B is Boltzmann's constant (see [1], page 600 for the definition of entropy). The free energy of the system is then given by

	$G = \sum_s N_s U_s + k_B T \sum_s N_s \, \mathrm{Ln}\left(N_s / N\right) + \sum_c n_c \mu_c$	Equation s26

where U_s is the internal energy of the transporter in state *s* (measured in Joules per molecule) and the first sum is the total internal energy of all the transporters in the system.

The chemical potential of a chemical species, *c*, is defined as

	$\mu_c = \dfrac{\partial G}{\partial n_c}$	Equation s27

which is consistent with the presence of μ_c in equation 1. The free energy is measured in Joules, the quantity (denominator in equation s27) is measured in moles and hence the chemical potential has dimensions of Joules/mol. In analogy, the chemical potential of the transporters in states, *s*, is given by

	$\mu_s = \dfrac{\partial G}{\partial n_s} = N_A \dfrac{\partial G}{\partial N_s}$	Equation s28

Where N_A is Avagardo's number and converts from molecules to moles. Applying equation s28 to equation s23 gives the chemical potential of the transporters in state *s* as

	$\begin{aligned} \mu_s &= N_A U_s + RT \, \mathrm{Ln}\, P_s \\ &= \mu_s^0 + RT \, \mathrm{Ln}\, P_s \end{aligned}$	Equation s29

Where P_s is the probability of finding a transporter in state *s* and is given by $P_s = N_s / N$ at any instant in time. By analogy with the chemical potential of a chemical species (equation 2), standard conditions could be defined as $P_s = 1$ and then the internal energy of N_A transporters would be equal to the standard chemical potential, that is, $\mu_s^0 = N_A U_s$.

The change in free energy for a reaction, ΔG, is defined as

	$\Delta G = \dfrac{\partial G}{\partial \varepsilon}$	Equation s30

where ε is the reaction progression in moles and hence ΔG is measured in Joules/mol. For a reaction which takes the transporter from state 1 to 2 ($T_1 \rightarrow T_2$), then $\Delta G_{1\rightarrow 2}$ is equal to

	$\Delta G_{1\rightarrow 2} = \dfrac{\partial G}{\partial n_1}\dfrac{\partial n_1}{\partial \varepsilon} + \dfrac{\partial G}{\partial n_2}\dfrac{\partial n_2}{\partial \varepsilon}$	Equation s31

As $\partial n_1 / d\varepsilon = -1$ and $\partial n_2 / d\varepsilon = +1$, this gives

	$\Delta G_{1\to 2} = \mu_2 - \mu_1$	Equation s32

where μ_1 and μ_2 are the chemical potentials of the transporter in state 1 and state 2, respectively. Just as in chemical thermodynamics, the change in free energy is the difference between the chemical potential of the products and substrates of the reaction. Likewise, when a reaction involves uptake of a chemical species, C, (T_1+C→T_2), the change in free energy is given by

	$\Delta G_{1\to 2} = \frac{\partial G}{\partial n_1}\frac{\partial n_1}{\partial \varepsilon} + \frac{\partial G}{\partial n_2}\frac{\partial n_2}{\partial \varepsilon} + \frac{\partial G}{\partial n_c}\frac{\partial n_c}{\partial \varepsilon}$	Equation s33

and, as $\partial n_c/d\varepsilon=-1$, then

	$\Delta G_{1\to 2} = \mu_2 - \mu_1 - \mu_c$	Equation s34

And again, the change in free energy is the difference between the chemical potential of the products and the substrates of the reaction.

In molecular dynamics simulations, the free energy surface (FES), which has dimensions of Joules/mole, is defined as [2]:

	$FES_s = -RTLn(P_s)$	Equation s35

where the state, *s*, would be the coordinates of all the atoms. Molecular dynamic simulations occur at equilibrium (ΔG=0) thus combining equations s29, s32 and s35 gives

	$FES_1 - FES_2 = \mu_1^0 - \mu_2^0$	Equation s36

Where the subscripts 1 and 2 refer to two states. Thus the free energy surface and the standard chemical potential refer to the same potential to an arbitrary constant for a system at equilibrium. Equation 14 is an alternative formulation of Boltzmann's distribution because it can be rewritten as:

	$\frac{P_1}{P_2} = e^{-(\mu_1^0 - \mu_2^0)/RT}$	Equation 15

which reveals that the standard chemical potential is the more fundamental description than the free energy surface when a system is not at equilibrium. The standard chemical potential and free energy surface are entirely different from the free energy of the system (equation 1).

References

[1] P. Atkins, J. de Paula, R. Friedman, Physica Chemistry Quanta, Matter, and Change, Second Edition ed., Oxford university Press, 2014.

[2] O. Valsson, P. Tiwary, M. Parrinello, Enhancing Important Fluctuations: Rare Events and Metadynamics from a Conceptual Viewpoint, Annu Rev Phys Chem, 67 (2016) 159-184.
